# Tobacco use among medical students and surrogate control of tobacco in India

**DOI:** 10.4103/0970-2113.76291

**Published:** 2011

**Authors:** Virendra Singh

**Affiliations:** *Division of Allergy and Pulmonary Medicine, SMS Medical College, Jaipur, Rajasthan, India. E-mail: drvirendrasingh@yahoo.com*

Tobacco is a known health hazard and also a definite cause of death. It is strongly suspected as root of more than 25 diseases. Tobacco is responsible for killing about 5 million people in the world every year. It kills half of people who use it. Chronic obstructive pulmonary disease (23%) and cancer of respiratory tract (18%) are the most common causes of death due to tobacco-induced diseases.[1]

Despite such alarming dangers, tobacco is extensively used and many users are not yet convinced about its ill effects. “If tobacco is so harmful, why majority of doctors smoke?” is a common arguments pushed by smokers during discussion. It is a general perception that medical professional use tobacco quite commonly. But this perception is a myth. Study done by Chatterjee *et al*., getting published in this issue of the journal substantiates this aspect. It shows that the prevalence of tobacco use among medical college students (18%) is far less than that in students (44%) of other colleges.[2]

Though prevalence of smoking among medical students is comparatively less yet it is a matter of concern, since they are considered as mentors of health care in the society. It is the question of public message through them and not the quantum of their tobacco use. Smoking by one doctor is far more assuring about safety of tobacco than smoking by dozens of politicians or bureaucrats. Conversely, quitting tobacco even by a single doctor is far more inspiring to give-up tobacco than the same act by other professionals.

Today we have enough evidence that tobacco is a slow poison. Need of hour is that the medical students understand their social responsibilities and say ‘NO’ to tobacco. They should initiate and become vanguards of an anti-tobacco movement.

Regarding tobacco use in India, the recent order of Honorable Supreme Court is a welcome step. The Apex Court of India has restrained the use of plastic material in sachets of ‘gutka’, tobacco, ‘pan masala’ etc. (Order dated 07 Dec, 2010, in favor of public petition filed by Indian Asthma Care Society). Though it may not directly affect tobacco sale but the ban on plastic sachets will restrict the easy availability and long time durability of tobacco products. Hopefully, the possible alternate, paper or metal containers for tobacco products will reduce the ease of the availability and increase the cost of the product, compelling users to give up. Thus the Supreme Court orders may prove an excellent surrogate step to control tobacco use in the society.
